# The genesis and evolution of acute myeloid leukemia stem cells in the microenvironment: From biology to therapeutic targeting

**DOI:** 10.1038/s41420-022-01193-0

**Published:** 2022-09-26

**Authors:** Yongfeng Chen, Jing Li, Linglong Xu, Mihnea-Alexandru Găman, Zhenyou Zou

**Affiliations:** 1 Department of Basic Medical Sciences, Medical College of Taizhou University, Taizhou, Zhejiang 318000 China; 2 Department of Histology and Embryology, North Sichuan Medical College, Nanchong, Sichuan 637000 China; 3 Department of Hematology, Taizhou Central Hospital (Taizhou University Hospital), Taizhou, Zhejiang 318000 China; 4 Faculty of Medicine, “Carol Davila” University of Medicine and Pharmacy, 050474 Bucharest, Romania; 5 Department of Hematology, Centre of Hematology and Bone Marrow Transplantation, Fundeni Clinical Institute, Bucharest, Romania; 6 Brain Hospital of Guangxi Zhuang Autonomous Region, Liuzhou, Guangxi 545005 China

**Keywords:** Haematopoietic stem cells, Oncogenesis

## Abstract

Acute myeloid leukemia (AML) is a hematological malignancy characterized by cytogenetic and genomic alterations. Up to now, combination chemotherapy remains the standard treatment for leukemia. However, many individuals diagnosed with AML develop chemotherapeutic resistance and relapse. Recently, it has been pointed out that leukemic stem cells (LSCs) are the fundamental cause of drug resistance and AML relapse. LSCs only account for a small subpopulation of all leukemic cells, but possess stem cell properties, including a self-renewal capacity and a multi-directional differentiation potential. LSCs reside in a mostly quiescent state and are insensitive to chemotherapeutic agents. When LSCs reside in a bone marrow microenvironment (BMM) favorable to their survival, they engage into a steady, continuous clonal evolution to better adapt to the action of chemotherapy. Most chemotherapeutic drugs can only eliminate LSC-derived clones, reducing the number of leukemic cells in the BM to a normal range in order to achieve complete remission (CR). LSCs hidden in the BM niche can hardly be targeted or eradicated, leading to drug resistance and AML relapse. Understanding the relationship between LSCs, the BMM, and the generation and evolution laws of LSCs can facilitate the development of effective therapeutic targets and increase the efficiency of LSCs elimination in AML.

## Facts

- Abnormal BM niche may be involved in the pathogenesis of AML.
- The BMM exerts a selective pressure for the clonal evolution in leukemia, and oxidative stress may represent another one.
- The protection from BMM and the clonal evolution of LSC may responsible for drug resistance in leukemia.
- Target BMM may be a potential therapeutic strategy for leukemia treatment.

## Open questions

- What is the exact role and mechanism of BMM in leukemogenesis?
- How to avoid damage to normal hematopoietic and hematopoietic supporting cells while eradicating leukemia cells?
- How to effectively repair the abnormal BMM?
- How to effectively unblock the protection of BMM on AML cells?

## Introduction

Acute myeloid leukemia (AML) is a group of highly heterogeneous clonal diseases. The constant accumulation of acquired somatic mutations in hematopoietic stem cells (HSCs) is the primary pathogenic mechanism of AML. Recently, the emergence of novel chemotherapeutic drugs and the progress of HSC transplantation have considerably improved the remission rate and disease-free survival of AML patients. However, the high relapse rate of AML, which is largely attributed to the residual leukemic stem cells (LSCs), remains an unsettled thorny issue. LSCs represent a subpopulation of malignant cells with unlimited self-renewal capacity and multi-directional differentiation potential. Despite representing a small proportion of all leukemic cells (approximately 0.1–1%), LSCs are considered the origin of leukemia and the primary cause of relapse after treatment, as well as of drug resistance [1]. LSCs are closely related to the bone marrow microenvironment (BMM). The BMM not only provides a habitat for LSCs but also offers the support for the survival and development of LSCs while alleviating the pressure imposed by chemotherapy on LSCs. This feature of the BMM can lead to the generation of dominant clones, resulting in leukemia relapse and drug resistance. Therefore, an in-depth investigation of the relationship between LSCs and the BMM, as well as the generation and evolution laws of LSCs, will facilitate the understanding of the pathogenesis and drug resistance mechanism of AML.

## Abnormal BM niche involvement in the pathogenesis of AML

The BM is the principal hematopoietic organ consisting of HSCs and the hematopoietic microenvironment. The latter is also known as the stem cell niche, which consists of bone marrow stromal cells (BMSCs) and extracellular matrix (ECM). Normal hematopoiesis is the process whereby HSCs self-renew and the progenitor cells proliferate and differentiate in the BMM [2].

BMSCs are integral components of the hematopoietic microenvironment and play an indispensable role in maintaining the stability, homing, proliferation, and differentiation of HSCs [3]. Accumulated evidence demonstrates that the dysfunction of BMSCs impairs the BMM, thereby affecting normal hematopoiesis. Besides, the dysfunction of BMSCs will trigger and promote the development of malignant hematological diseases. Many studies have reported that AML is linked with a reduction in BMSCs. In most AML cases, cytogenetic abnormalities of BMSCs are usually found [4–7]. Besides, BMSCs derived from AML patients may exhibit molecular and functional alterations, e.g., higher senescence, decreased proliferation, reduced clonogenic potential, impaired in vitro osteogenic and adipogenic differentiation, imbalanced regulation of endogenous hematopoiesis, and increased support of leukemia growth [8–11].

Recently, several research groups have used animal models to explore the role of BMM in leukemia. According to Raaijmakers et al., selectively knocking out Dicer1 (a gene required for RNA and microRNA processing) in mesenchymal osteoprogenitors impaired osteoblast differentiation both in vitro and in vivo. In mouse models, the loss of Dicer1 in osteoprogenitors induced an evident hematopoietic disorder, increased apoptosis, and proliferation of hematopoietic stem and progenitor cell (HSPC). These knockout animals exhibited key features of human myelodysplastic syndromes (MDS), including the propensity to develop AML [12]. A study by Kode et al. demonstrated that activating mutations of β-catenin in mouse osteoblasts stimulated the expression of Notch ligand jagged 1, which subsequently altered the differentiation potential of myeloid and lymphoid progenitors through the activation of Notch signaling, and ultimately led to the development of AML [13]. These findings suggested that functional abnormalities caused by genetic mutations in BMSCs may play a crucial role in leukemogenesis. Yilmaz et al. implanted HSCs with tumor suppresser gene phosphatase and tensin homolog (PTEN) knocked out into irradiated recipient mice. These PTEN-deficient HSCs gradually depleted over time after a brief multilineage differentiation, while no evidence of myeloproliferation or development of leukemia was discovered in any recipient mice. In contrast, the implantation of PTEN-deficient whole bone marrow cells led to myeloproliferative progression to leukemia/lymphoma. These results suggested that BMSCs may play a more critical role in the malignant transformation of normal hematopoietic cells into leukemia cells [14]. The occurrence of donor cell leukemia in clinical AML cases further indicates that the hematopoietic microenvironment may be involved in the pathology of AML. However, the concrete mechanism underlie the hematopoietic microenvironment deterioration is still poorly understood. Recent evidence suggested that oxidative stress may play a key role: Huang et al. investigated the effect of iron overload (IO) on BMSCs in MDS and AML patients. The results showed that IO could promote the ROS generation and apoptosis in BMSCs, decrease expression of hematopoietic regulation-related genes such as VEGFA, CXCL12, and TGF-β1, and activate the Wnt/β-catenin signaling pathway, and it was supposed that these alterations may be associated with the MDS progression [15]. Li et al. showed that daunorubicin (DNR) treatment led to senescence of mouse BMSCs, increased ROS production, as well as mitochondrial dysfunction. When oxidatively damaged BMSCs were co-cultured with HSCs in vitro, these stromal cells generated more ROS, leading to increased genomic instability in HSCs [16]. In addition, BMSCs injury might also lead to impaired immune surveillance and dysregulated cytokine secretion, disrupted BMM homeostasis, and ultimately led to abnormal proliferation of hematopoietic cells, all of which may be potential causes of malignant transformation of hematopoietic cells [16]. However, the exact role and mechanism of BMM in leukemogenesis still need to be elucidated by more extensive and in-depth studies.

## Origins and generation of LSC

It is generally believed that LSC result from mutation accumulation in HSC. Long-term HSCs are capable of self-renewal. Long-term exposure to carcinogens will cause the accumulation of genetic mutations in these HSCs, increasing the risk of malignant transformation of these cells. Hope et al. performed continuous tracking of the transplanted LSCs and highlighted that LSCs are not functionally homogeneous but, similarly to the normal HSC compartment, comprised distinct hierarchically arranged LSC classes. This finding supported the hypothesis that LSCs originate from normal HSCs [17]. In another study, Cozzio et al. adopted transgenic technology to analyze three types of cells with different differentiation grades: HSCs, common myeloid progenitors, and the lineal descendent granulocytic/monocytic-restricted progenitors. These three types of cells could survive and proliferate in vitro after mixed lineage leukemia (MLL) fusion genes were introduced. The mice could be successfully transfected with these cells, which induced leukemia at the same speed [18]. Thus, it was speculated that LSCs have a wide variety of sources. LSCs may be derived from HSCs and progenitor cells that are earlier in the development stage and also from leukemia progenitor cells that are more mature.

According to the AML two-hit model, two major types of genetic mutations play a pivotal role in the pathogenesis of AML. Type I genetic mutation involves the tyrosine kinases in the signal transduction pathway, including the signaling molecules FMS-like tyrosine kinase-3 (FLT3), stem cell factor receptor (c-Kit), and break point cluster region-abelson (BCR-ABL). Persistently activated tyrosine kinases confer special benefits for the proliferation and survival of hematopoietic precursor cells [19]. Type II mutation involves disrupting transcription factors or transcriptional coactivators, such as acute myeloid leukemia-1 transcription factor/eight-twenty-one corepressor (AML1/ETO), mixed-lineage leukemia/ALL1-fused gene from chromosome 9 protein (MLL/AF9), and promyelocytic leukemia/retinoic acid receptor α (PML/RARα) fusion gene. These abnormal changes can lead to disorders of differentiation, maturation, and apoptosis of hematopoietic progenitor cells (HPC) [20]. In mouse models, when genetic abnormalities of type I occurred alone, chronic myeloid leukemia (CML)-like changes were noted; when genetic abnormalities of type II occurred alone, MDS-like changes were noted. Under normal situations, leukemia occurs when these two types of genetic abnormalities occur simultaneously [21]. So far, it remains uncertain how the two types of genetic abnormalities work synergistically.

Recently, a large number of studies have shown that AML patients carry repeated mutations of many important epigenetic mediators. Among them, mutations of genes related to DNA methylation are most common, including DNA methyltransferase 3A (DNMT3A) and isocitrate dehydrogenase 1 and 2 (IDH1/2) mutations. Genetic modeling of individual epigenetic factors has shown that most of these mutations will affect the HSC compartment and alter hematopoietic differentiation, in some cases leading to myelodysplasia, stem cell expansion, or other preleukemic conditions [22–24]. Besides, mutations of other epigenetic modifiers, such as additional sex combs-like (ASXL1) and enhancer of zeste homolog 2 (EZH2), also play an important role in the occurrence of AML (Table 1). However, epigenetic mutations alone are not sufficient to transform HSCs, indicating that sequential acquisition of mutations is required [25]. In a recent study, Uckelmann et al. reported that in Npm1c/Dnmt3a mutant knock-in mice, a model of AML development, the use of a small molecule (VTP-50469) can reverse the self-renewal of myeloid progenitor cells before leukemia transformation. This finding indicates that individuals at high risk of developing AML might benefit from targeted epigenetic therapy in a preventative setting [26].

**Table 1:** Common epigenetic modifier mutations in AML.

Classification	Modifier	Biological activity	Frequency	Ref.
DNA methylation	DNMT3A	Majority of DNMT3A mutations are heterozygous missense mutations causing premature truncation of R882. The interaction between R882 and PRC1 leads to downregulation of hematopoietic differentiation genes, inducing aberrant proliferation of HSPC.	12–35%	[104, 105]
	TET2	TET2 catalyzes the conversion of 5mc to 5-hmC, resulting in demethylation. Loss of TET2 function can increase methylation and reduce the expression of mitotic checkpoint proteins MAD2 and CDC20, leading to CIN.	10%	[104, 106]
	IDH1/2	The interaction between IDH and TET2 leads to increased methylation and impaired DNA damage repair functions.	20%	[104]
	MLL	The most common form of MLL gene rearrangement is chromosomal translocation, which is usually the translocation fusion between MLL-N and the C-terminal domain of translocation partner gene (TPG) to form fusion genes. The formation of MLL fusion protein induces overexpression of HOXA9 and Meis1 genes, which contributes to the over proliferation of immature HSPC.	5–10%	[107, 108]
Histone modification	EZH2	EZH2 controls expression of genes involved in stem cell maintenance and differentiation. Down-regulation of EZH2 inhibited apoptosis, affected MAD2 and CDC20 expression, and promoted CIN in AML cells.	4%	[106, 109]
	CBP	CBP alterations lead to HAT inactivation, which may result in faulty histone acetylation and abnormally activated gene expression, promoting leukemia transformation.	2.5%	[110–112]
	HDAC	HDAC antagonizes the acetylation of HAT and inhibits the expression of tumor suppressor genes, while the down-regulation of HDAC can enhance the activity of some tumor suppressor genes and promote their transcription and translation.	2%	[113, 114]
	ASXL1	ASXL1 mutations resulted in increased stabilization of BAP1 and its recruitment to chromatin and the induction of an oncogenic transcriptional program.	20%	[104, 115]

Abbreviations: *5mC* 5-methylcytosine, *5-hmC*, 5-Hydroxymethylcytosine, *ASXL1* Additional sex comb-like 1, *BAP1* BRCA1-associated protein 1, *CBP* CREB-binding protein, *CDC20* Cell-division cycle protein 20, *CIN* Chromosome instability, *EZH2* Enhancer of zeste homolog 2, *HAT* Histone acetyltransferases, *HDAC* Histone deacetylases, *HOXA9* Homeobox A9, *MAD2* Mitotic Arrest Deficient 2, *Meis1* Meis homeobox 1, *PRC1* Polycomb Repressive Complex 1, *TET2* Ten-eleven translocation methylcytosine dioxygenase 2.

## Evolution of LSC

Recent studies have shown that AML cell populations at relapse may have evolved from either the dominant clonal or minor subclonal cell populations present at diagnosis, accompanied by the potential acquisition of additional mutations. AML cells adapt to the environment by steady, continuous clonal evolution and better survive under chemotherapy pressure [27–30].

The clonal evolution in leukemia is a multi-step, dynamical evolutionary process. Corces-Zimmerman et al. analyzed the persistence of preleukemic mutations in patients in remission. It was discovered that mutations in preleukemia were present in CD34^+^ progenitor cells and various mature cells, indicating that preleukemic HSCs can survive the induction chemotherapy, thereby confirming these cells as a reservoir for the reevolution of relapsed disease [31]. Moreover, they tentatively proposed several patterns of clonal evolution leading to AML relapse based on the existing whole genome sequencing results: (1) treatment refractory primary disease, (2) further evolution of a dominant clone present at diagnosis, (3) outgrowth of a subclone present at diagnosis; (4) further evolution of disease from a preleukemic HSC [31]. Miles et al. employed single-cell DNA sequencing to analyze the samples from 123 patients with myeloid malignancies to determine the clonal features of myeloid malignancies. The results showed that AML is dominated by a small number of clones. Among different types of genetic mutations, epigenetic mutations usually occur at the early stage of leukemogenesis, and these mutations tend to occur simultaneously. In contrast, mutations of genes related to the cell signaling pathways occur much later, which are mainly found in distinct subclones, consistent with increased clonal diversity. Besides, it was found that increased clonal diversity in AML did not coincide with the difference in the number of mutations within the largest clone, suggesting that increased mutational burden within a clone is not the primary driver of clonal dominance [32].

**Fig. 1:**
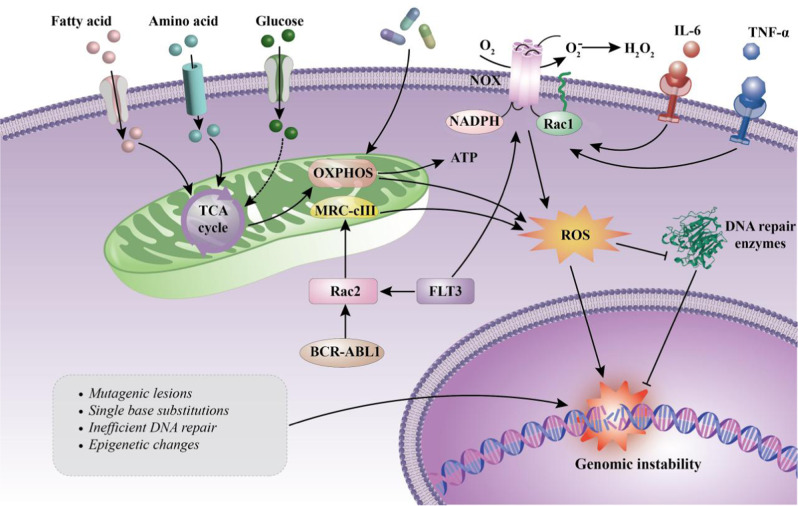
ROS exert selective pressure for the clonal evolution in AML. Different from HSC, which mainly obtains energy through glycolysis, LSC mainly relies on oxidative phosphorylation (OXPHOS) to support cell metabolism and survival, thus producing a relatively high ROS level [94]. Chemotherapeutic drugs and chronic inflammation also promote ROS production [37, 41, 42]. In addition, oncogenes such as FLT3(ITD) and BCR-ABL1 can also facilitate intracellular ROS production through NOX or RAC2-MRC cIII pathway [95, 96]. High levels of ROS not only lead to mutagenic reactions in the DNA, but also inhibit DNA repair enzymes, resulting in genomic instability, which may be an important driver of LSC evolution [97]. Rac Rac GTPase; TCA tricarboxylic acid; MRC-cIII mitochondrial respiratory chain complex III.

A major distinctive feature of cancer cells is their genetic instability. Cancer cells may evolve along paths under different selective pressures to gain survival and development advantages, a primary reason for continuous clonal evolution in leukemia. The BMM exerts a selective pressure for the clonal evolution in leukemia. It is not only a shelter for leukemic cells but also supports the survival and development of leukemia cells by interacting with them, thereby relieving the chemotherapeutic pressure and promoting the generation of dominant clones (see section 4). Elevated reactive oxygen species (ROS) levels are hallmark features of leukemia cells [33, 34], and oxidative stress induced by high level of ROS may represent another selective pressure for the clonal evolution in leukemia. The sources of ROS levels in AML cells are complex and diverse. Among them, nicotinamide adenine dinucleotide phosphate (NADPH) oxidase (NOX) and mitochondrial electron transport chain (mtETC) are considered to be the main sources of ROS in leukemia cells [35]. However, Hole et al. found that AML cells treated with NOX inhibitors could more effectively inhibit the production of superoxide compared with mtETC inhibitors and mitochondrial ROS scavengers, therefore suggesting that ROS in AML cells is primarily derived from NOX [36]. It was reported that, oncogenic kinases like BCR-ABL1, FLT3-internal tandem duplication (ITD), or c-KIT can promote the generation of endogenous ROS by increasing the activities of RAC GTPases or membrane-bound NOX [37–40]. In addition, chemotherapy and chronic inflammation can also increase ROS generation, thereby elevating oxidative stress [37, 41, 42]. Although quiescent AML LSCs have a strong ability to maintain the redox balance, they still suffer from oxidative damage to DNA caused by high ROS levels. Such damage increases genomic instability and drives the genetic evolution of LSCs under the synergistic action of the leukemic microenvironment, from which LSCs gain a competitive advantage [35, 43] (Fig. 1). Further investigations are needed to reveal the specific mechanism by which LSCs evolve genetically.

## Interactions between AML cells and the BMM

**Fig. 2:**
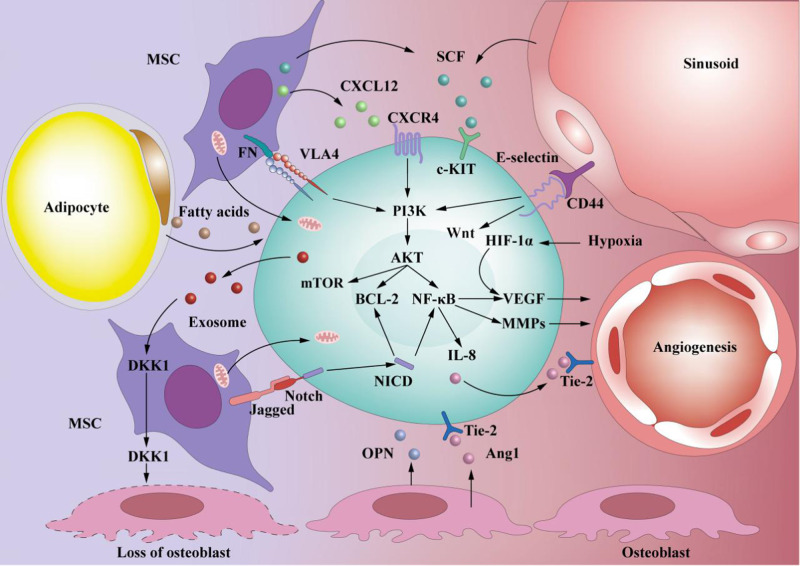
Interaction between LSC and BMM. LSC interact and adhere to various niche cells (such as MSCs, osteoblasts, adipocytes, and endothelial cells) and various ECM molecules secreted by them. The interaction between LSC and BMM can activate many important signaling pathways, thereby regulating the biological function of LSC and remodeling BMM accordingly. (1) The CXCL12/CXCR4 axis plays a key role in LSC maintenance and can activate multiple signaling pathways, such as PI3K/AKT/mTOR, to regulate the survival and proliferation of LSC [98]; (2) The interaction between VLA4 from LSC cells and fibronectin (FN) from MSCs activates the PI3K/AKT/BCL2 pathway, allowing LSCs to be resistant to cytotoxic drugs [99]; (3) The binding of E-selectin to CD44 activates the Wnt [100] and PI3K/AKT/NF-κB signaling pathway [101], and promotes LSC survival; (4) The binding of Jagged to Notch activates the Notch signaling pathway, and the intracellular domain NICD of Notch is then released and translocated into the nucleus to activate the transcription of related genes [102]; (5) Hypoxia can promote the HIF-1α-VEGF signaling pathway and angiogenesis. In addition, NF-κB can promote the production of MMPs and VEGF, which in turn accelerates angiogenesis [103]; (6) LSC-secreted exosomes can induce the expression of DKK1 in MSCs, a suppressor of normal hematopoiesis and osteogenesis, thereby leading to the loss of osteoblasts [50]. BCL-2 B-cell lymphoma 2; Fn fibronectin; MMPs matrix metalloproteinases; mTOR mammalian target of rapamycin; NF-κB nuclear transcription factor-κB; OPN osteopontin; VLA4 very late antigen 4; Wnt wingless-type protein.

The interactions between LSCs and the microenvironment are fundamental to survival, therapeutic resistance, and relapse of leukemia. In the BMM, hypoxia induces the synthesis of hypoxia-inducible factor-1α (HIF-1α), which plays a key role in cell migration and survival. In human AMLcells, HIF-1α knowdown leads to increased apoptosis and impaired cell engraftment [44]. Furthermore, hypoxia can also upregulate C-X-C chemokine receptor type 4 (CXCR4) expression in AML cells, the cognate receptor of chemokine ligand 12 (CXCL12), and promote CXCL12 expression in BMSCs *via* HIF-1α [45, 46]. Relying upon the interaction between CXCR4 and CXCL12, as well as the adhesion effect mediated by other surface adhesion molecules, AML cells dwell in the BM niches to maintain their survival [44–47]. Besides, the interaction between BMM and AML cell can also lead to the activation of phosphatidylinositol-3-kinase/protein kinase B (PI3K/AKT), wingless-related integration site (Wnt), NOTCH, and other signal pathways, which play key roles in the regulation of AML cells’ maintenance, proliferation, and differentiation (Fig. 2). A series of in vitro and in vivo experiments confirmed that AML cells can remodel the BMM by inducing the abnormal proliferation and differentiation of BM mesenchymal stem and progenitor cells (MSPCs), thereby favoring disease development and progression [48, 49]. Evidence suggests that AML cell-derived exosomes can stimulate the expression of Dickkopf-1 (DKK1), thereby inducing downregulation of HSC-supporting factors in BMSCs and reducing their ability to support normal hematopoiesis [50]. Furthermore, AML cell-derived exosomes can also downregulate critical retention factors, such as stem cell factor (SCF) and CXCL12, leading to the mobilization of HSPC out of the BM [51]. In a recent study, Scoville et al. found that human AML blasts can activate the transcription factor aryl hydrocarbon receptor (AHR) pathway and induce miR-29b expression in natural killer (NK) cells, thereby impairing NK cells maturation and function, which may be one of the important mechanisms underlying AML cells’ immune escape [52].

In severely hypoxic BM tissues, leukemic cells exhibit the strong adaptive ability and promote angiogenesis *via* multiple mechanisms. Such a capacity is needed to meet the demand for oxygen and nutrients in rapidly growing leukemic cells. Hypoxia has been reported to promote AML cells’ upregulation of transcription and expression of several angiogenic factors, such as vascular endothelial growth factor (VEGF), thus increasing the angiogenic activity of endothelial cells [53, 54]. Angiopoietin/Tyrosine Kinase With Immunoglobin-like And Epidermal Growth Factor-like Domain-2 (Ang/Tie-2) axis is another signaling pathway that is significantly associated with angiogenesis [55]. Tie-2 receptor is always overexpressed in AML cells, which promotes angiogenesis *via* interaction with Ang-1 [56]. The Ang-1/Tie-2 interactions also facilitates the adhesion of AML cells to BM niche, and helps to maintain the quiescent and anti-apoptotic state of LSC in BM niche [57, 58] (Fig. 2). In vitro experiments showed that Tie-2-blocking antibodies had a growth inhibitory effect on human AML cells co-cultured with microvascular endothelial cells [58]. However, the application of antiangiogenic drugs is often found ineffective in clinical studies, indicating that the deterioration of BMM is not simply due to angiogenesis. Recently, Passaro and colleagues reported that in human AML patient-derived xenografts, significant abnormalities in the bone marrow vasculature were observed. The engrafted AML not only promoted angiogenesis but also increased the permeability of blood vessels. Further analysis showed that the increased vascular permeability was associated with hypoxia-induced increases in ROS and NO. Cytarabine (AraC) treatment achieved only transient remission, and it was supposed that this may be relevant to the increase of vascular permeability, since increased vascular permeability not only leads to poor drug delivery, but also favors the tumor growth and enhances metastatic potential. The combination of nitric oxide synthase inhibitors and chemotherapy successfully restored normal vasculature and delayed leukemia, leading the way to combine leukemia-niche therapies in clinical trials [59].

Fatty acid metabolism is the main metabolic pathway for AML cell survival in the adipocyte-rich BMM [60]. The study by Shafat et al. found that AML cells can alter adipocyte metabolic processes, inducing lipolysis of triglyceride to fatty acid, and the fatty acid was then transferred to AML cells to fuel FAO, thereby promoting the survival and proliferation of AML cells [61]. Tunneling nanotubes (TNTs), or membrane nanotubes, are an important means of communication between eukaryotes [62]. Recent studies have shown that AML cells propel mitochondrial transfer from BMSCs to AML cells *via* TNTs under the stimulation of ROS, which generated from NOX. As a result, more energy is generated for survival of AML cells by mitochondrial OXPHOS. However, such a phenomenon was not observed in HSCs that derive energy primarily from anaerobic glycolysis [63]. These studies demonstrated that AML cells acquire conspicuous survival benefits by interacting with BMSCs.

## Targeted therapy for AML

LSCs usually reside in a quiescent state, that is, the G_0_ phase, when the LSCs replicate slowly but have an unlimited self-renewal capacity. This biological characteristic of LSC make it insensitive to the cytotoxicity of the conventional cell cycle-targeting chemotherapy. Besides, the expression of multi-drug resistance proteins in LSCs results in poor clinical outcomes. In recent years, the advancement of both biomedical technology as well as the understanding of AML drug resistance mechanisms strongly promote the development of AML-targeted drugs. Drugs targeting AML cell surface antigens, mutant genes, and intracellular signaling pathways have become the current research trend and focus (Table 2), while the exploration of new targets is also ongoing. NOX is a major source of pro-survival ROS in AML cells and is therefore an ideal target to inhibit pro-survival signaling in AML cells. Among NOX family members, NOX1, NOX2, and NOX4 are expressed in human CD34^+^ hematopoietic progenitor cells [64, 65]. NOX2 inhibitors have been reported to induce reduction of intracellular ROS in FLT3-ITD AML cells, inhibition of downstream growth and survival pathways of FLT3, and increased apoptosis associated with induction of mitochondrial ROS and restoration of p38^MAPK^ [66]. Setanaxib, an inhibitor with anti-NOX1 and NOX4 functions, has anti-proliferative effects on both FLT3-ITD-positive and negative AML cells, and has strong synergistic effects with cytotoxic drugs anthracyclines such as daunorubicin. This anti-proliferative effect may be mediated by amplification of oxidative stress signaling by promoting ROS production from sources other than NOX [67]. It was reported that a variety of chemotherapeutic drugs such as daunorubicin, cytarabine, and decitabine can also induce apoptosis in leukemia cells *via* ROS generation [68–70]. However, high levels of ROS inevitably cause oxidative damage to hematopoietic and hematopoietic supporting cells while eliminating leukemia cells.

**Table 2:** Ongoing clinical trials evaluating novel targeted agents for AML.

T	NCT	Drug	Target	Patient population	Phase
Targeting the surface antigens	NCT03386513	IMGN632	CD123	Patients in whom CD123 can still be detected after receiving CD123 targeted drug treatment.	Phase 2
	NCT04342962	Tagraxofusp	CD123	CD123^+^ relapsed adult AML patients	Phase 2
	NCT03867682	Lintuzumab	CD33	Adult AML patients	Phase 2
	NCT04435691	Magrolimab	CD47	Adult AML patients	Phase 2
	NCT03647800	APVO436	CD123×CD3	Adult AML and MDS patients	Phase 1
	NCT04582864	Flotetuzumab	CD123×CD3	Relapsed adult AML and MDS patients	Phase 2
	NCT03224819	AMG673	CD33×CD3	Adult R/R AML patients	Early phase 1
Targeting the mutant genes	NCT05024552	Gilteritinib	FLT3	Adult R/R AML subjects with FLT3 mutations	Phase 1
	NCT03258931	Crenolanib	FLT3	Newly diagnosed AML subjects with FLT3 mutations	Phase 3
	NCT03793478	Quizartinib	FLT3	Pediatric R/R AML subjects with FLT3-ITD mutations	Phase 1
	NCT03573024	Azacitidine	DNMT3A	Non-elderly adult patients with AML	Phase 2
	NCT03844815	Decitabine	DNMT3A	Adult AML patients	Phase 1
	NCT04493164	Ivosidenib	IDH1/2	Adult AML patients with IDH1 mutations	Phase 2
	NCT04203316	Enasidenib	IDH1/2	2–18 years R/R AML patients	Phase 2
	NCT03843528	Vorinostat	HDAC	Childhood myeloid malignancies	Phase 1
Targeting the intracellular signaling pathways	NCT04173585	Bortezomib	NF-κB	Adult R/R AML patients	Phase 2
	NCT04655391	Glasdegib	Hedgehog	Adult patients with relapsed AML post alloHCT	Phase 1
	NCT04562792	Daunorubicin	Wnt	1–21 Years R/R ALL and AML patients	Phase 2

*alloHCT* allogeneic hematopoietic cell transplantation, *ALL* acute lymphoblastic leukemia, *MDS* myelodysplastic syndrome, *R/R* Relapsed/Refractory.

Targeting the weaknesses in the metabolism of leukemia cells has attracted much attention in recent years. Fatty acid metabolism is the main metabolic pathway for AML cells to maintain survival, and drug-resistant LSCs have a higher FAO rate, suggesting that FAO is related to the drug resistance of LSCs [71]. However, intervention using the FAO inhibitor-avocatin-B was ineffective. Further investigation showed that glucose uptake and glycolysis were increased while inhibiting the FAO in AML cells, which promoted AML cell survival [60]. This finding suggested that targeting a single metabolic pathway has limitations as leukemic cells may escape through metabolic adaptation. Similarly, although the combination of Bcl-2 inhibitor venetoclax and azacitidine (ven/aza) inhibited OXPHOS in vivo and resulted in an effective eradication of LSCs [72, 73], ven/aza failed to eradicate LSCs in relapsed/refractory (R/R) patients, suggesting that metabolic properties had altered. Metabolomic analysis revealed that nicotinamide metabolism was elevated in relapsed LSCs, which activated amino acid metabolism and fatty acid oxidation to drive OXPHOS, and therefore avoided the cytotoxicity of ven/aza treatment [74, 75]. Recently, several research teams started to use nicotinamide phosphoribosyltransferase (NAMPT) as a new intervention. NAMPT is the rate-limiting enzyme in nicotinamide metabolism, and its application can selectively eradicate R/R LSC [74, 76]. These findings suggested that targeting metabolic weaknesses in AML cells may be an effective strategy.

Hypoxia is an important feature of the leukemia microenvironment and also an ideal target in AML therapy. In the hypoxic environment, the maintenance of neutral pH represents a key survival mechanism for leukemic cells. In the hypoxic environment, Carbonic Anhydrases IX and XII (CA IX/XII) function as transmembrane proteins that mediate intracellular pH value regulation, which is critical for leukemic cell survival, because maintaining a neutral pH represents a key survival mechanism for tumor cells in hypoxia [77]. The study by Chen et al. recently showed that dual CA IX/XII inhibitor FC531 can significantly reduce the pH level and induce apoptosis in AML cells in vitro with a synergistic effect with cytarabine. Furthermore, FC531 exhibited anti-leukemia effect in single-agent mode or in combination with cytarabine in vivo, which significantly improved the survival rate of AML mice [77]. TH-302 is a 2-nitroimidazole-linked prodrug that can be activated under hypoxia to generate potent cytotoxin bromo-isophosphoramide mustard (Br-iPM). In vitro experiments showed that TH-302 could inhibit the proliferation of AML cells and promote cell apoptosis by reducing the expression of HIF-1α. Additionally, in vivo experiments demonstrated that TH-302 could effectively prevent disease progression in AML mice and prolong mouse life expectancy [78, 79]. Moreover, TH-302 showed synergistic anti-leukemia effects in the FLT3-ITD AML model when combined with the tyrosine kinase inhibitor - Sorafenib [79]. It should be emphasized that the protection and repair of BMM are as crucial as eliminating leukemia cells. In addition to the aforementioned repair of the BM vasculature, the restoration of redox balance is also a hot topic of current concern. Theoretically, scavenging excessively high levels of ROS with antioxidants should foster the repair of BMM, thereby boosting the restoration of normal hematopoiesis. However, the use of antioxidants is not recommended clinically because malignant cells may also be protected. Several recent studies have shown that a variety of natural compounds, such as curcumin, quercetin, and resveratrol could protect normal cells from oxidative damage, exhibited excellent anti-leukemia effects, and synergized well with chemotherapeutic drugs, suggesting that natural antioxidants may have broad application prospects in clinical adjuvant therapy [80–84]. Notably, malignant niches are frequently coupled with inflammatory responses, however, the impact of inflammation on hematopoietic microenvironment homeostasis is poorly understood. Recently, an in vivo and in vitro study by Habbel et al. confirmed that AML cells could secrete inflammatory cytokines and activate the Janus kinase/signal transducer and activator of transcription (JAK/STAT) signaling pathways in both AML blasts and BMSCs, thereby increasing the ROS generation and AML proliferation. Blockade of this inflammatory response signaling pathway resulted in the suppression of AML cells, suggesting that improving the hematopoietic microenvironment by inhibiting the inflammatory response is essential to amplify the anti-leukemia effect [85]. Blocking the connection between AML cells and BMM and unblocking the protection of BMM on AML cells are both critical for eliminating leukemia cells. It was demonstrated that the application of anti-CD44, anti-CD82, and anti-CD98 monoclonal antibodies could block the adhesion between the microenvironment and AML cells, thereby inhibiting the survival of AML cells [86–88]. The CXCL12/CXCR4 axis is an important mechanism regulating the interaction between the microenvironment and AML cells. CXCR4 inhibitors, such as plerixafor, BL-8040, and LY2510924, could disrupt the chemotaxis mediated by the CXCL12/CXCR4 axis and dislodge the leukemic cells from their protective BM niches to increase the sensitivity of leukemia cells to chemotherapy [89–91]. However, one major defect of inhibiting CXCL12-CXCR4 interaction is the release of a large number of leukemic cells into the peripheral blood. In that case, the potential of leukemic cells infiltrating extramedullary organs will be enhanced. At present, nearly all open-label clinical trials using CXCR4 inhibitors as chemosensitizers involve the combination of the CXCR4 inhibitors and different chemotherapeutic drugs to increase the possibility of the activated leukemic cells being killed [92, 93].

## Conclusion

BMM is not only the place where LSCs are generated, but also serves as a sanctuary for these malignant cells and provides support for their clonal evolution, reinforcing its involvement in the occurrence of drug resistance and recurrence of leukemia. Drug resistance mechanisms of leukemia mediated by BMM are highly complex. Various factors, including the adhesion between the leukemic cells and the microenvironment, signal transduction, and gene expression regulation, are involved, which form a complex regulatory network. The key components of the network may be new targets for leukemia treatment. Therapies targeting the interactions between BMM and leukemic cells are new strategies in the management AML, which are also the hotspots for research on the drug resistance mechanism of leukemia. However, the existing studies have some limitations, i.e., most are in vitro studies and cannot fully mimic the BMM under pathological conditions. Neither can such studies realistically reproduce the clonal evolution of leukemia in the BMM. Therefore, it is hard to precisely depict the real inner relationship between clonal evolution, heterogeneity, and drug resistance of AML. Another urgent problem is that the selectivity of targeted drugs needs to be further improved. BMM harbors leukemic cells and normal HSCs as well. Targeted drugs with poor selectivity may adversely affect the protective effect of the BMM on HSCs or even cause direct harm to HSCs, resulting in adverse reactions such as BM suppression. Therefore, constant optimization and more efforts are necessary in these fields. It is believed that in the near future, with the emergence of more perfect leukemia models and the development of new generation of analysis technology, the law of leukemia occurrence and development will be more deeply recognized, thus facilitating the precise treatment of leukemia.

## Data Availability

Data sharing not applicable to this article as no datasets were generated or analyzed during this study.
